# Post-infectious Cytomegalovirus Rhombencephalitis in an Immunocompetent Adult: A Case Report

**DOI:** 10.7759/cureus.96344

**Published:** 2025-11-07

**Authors:** Mustafeez Ur Rehman, Hadia Saeed

**Affiliations:** 1 Internal Medicine Department, Manchester University NHS Foundation Trust, Manchester, GBR; 2 Emergency Department, King's College Hospital NHS Foundation Trust, London, GBR

**Keywords:** brainstem encephalitis, cerebrospinal fluid analysis, cytomegalovirus, magnetic resonance imaging, postinfectious complication, rhombencephalitis

## Abstract

Cytomegalovirus (CMV) is a ubiquitous virus that typically causes subclinical infection in immunocompetent hosts. Neurological complications, particularly encephalitis, are exceedingly rare in this population. We present a case of a previously healthy young man who developed an acute brainstem syndrome characterised by dysarthria, ataxia, and altered consciousness. Early investigations were inconclusive, with bland cerebrospinal fluid (CSF) findings despite raised opening pressure and normal initial MRI. Repeat imaging revealed a subtle medullary lesion, while serology demonstrated primary CMV infection with detectable viraemia and negative CSF CMV polymerase chain reaction (PCR), consistent with a post-infectious mechanism. The patient made a steady, spontaneous recovery without antiviral therapy. This case highlights the diagnostic challenges posed by atypical presentations of CMV-associated rhombencephalitis in immunocompetent individuals, the value of repeat neuroimaging and serological testing, and the importance of multidisciplinary decision-making to avoid unnecessary treatment in selected cases.

## Introduction

Cytomegalovirus (CMV) is a ubiquitous beta-herpesvirus that infects the majority of adults worldwide and establishes lifelong latency within host cells [1]. The global seroprevalence of CMV is high, but the incidence of primary CMV infection in immunocompetent adults is estimated at approximately 10-50 cases per million seronegative individuals per year in developed countries, whereas symptomatic or severe CMV disease in previously healthy adults is considered extremely rare, with an estimated frequency of around one case per million persons annually. Although primary CMV infection in healthy individuals is typically asymptomatic or mild, reactivation in immunocompromised patients, including those with advanced HIV infection, post-transplant immunosuppression, or haematological malignancy, can cause severe neurological disease, including encephalitis, ventriculoencephalitis, and rhombencephalitis [2-4]. CMV encephalitis in this population carries significant morbidity and mortality despite antiviral therapy [5].

In contrast, CMV-associated central nervous system (CNS) disease in immunocompetent adults remains rare and diagnostically challenging. Over the last two decades, scattered case reports have highlighted presentations ranging from meningitis to life-threatening rhombencephalitis [6-9]. These cases frequently demonstrate normal or minimally abnormal cerebrospinal fluid (CSF) and subtle or delayed neuroimaging changes, which can delay diagnosis and lead to unnecessary or inappropriate treatments [10-12].

Rhombencephalitis is characterised by inflammation of the brainstem and cerebellum and presents with cranial neuropathies, ataxia, ophthalmoplegia, and altered consciousness. While classically associated with pathogens such as *Listeria *monocytogenes, CMV has emerged as an increasingly recognised, though still uncommon, etiology [11,13]. Importantly, its clinical and radiological features can overlap with post-infectious or immune-mediated syndromes, including Miller Fisher syndrome and Bickerstaff brainstem encephalitis [12,14,15].

We report a case of post-infectious CMV-associated rhombencephalitis in an otherwise healthy young man with bland CSF, initially normal MRI, and steady spontaneous recovery without antiviral therapy. This case underscores the importance of recognising atypical presentations of CMV CNS disease and highlights the role of repeat imaging, serological monitoring, and multidisciplinary decision-making in guiding management.

## Case presentation

A 25-year-old man with no significant past medical history presented initially to the emergency department with fever, headache, and dizziness. He had a recent history of tonsillitis and peri-oral ulceration and worked in pest control, with occupational exposure to rodents. At his first presentation, examination revealed horizontal nystagmus but was otherwise unremarkable, and he was discharged after a normal CT head scan.

He re-attended 21 hours after his initial visit with worsening drowsiness, slurred speech, diffuse myalgia, arthralgia, gait unsteadiness, urinary retention, and subjective limb weakness. On arrival, his Glasgow Coma Scale was 12-13. He was febrile and exhibited horizontal nystagmus, dysarthria, and variable lower-limb weakness with diminished reflexes. Neck flexion provoked pain, but there was no clear meningism.

He was treated empirically for meningoencephalitis with intravenous ceftriaxone, acyclovir, and metronidazole, and admitted for further investigations. Over the next 48 hours, he remained encephalopathic but not comatose, requiring close neurological monitoring. Intermittent areflexia and urinary retention suggested involvement of both central and peripheral pathways. Physiotherapy noted ataxia and impaired proprioception, though he remained able to mobilise with assistance.

Investigations

Initial bloods showed lymphocytosis and mild transaminitis (Table 1). Procalcitonin and C-reactive protein were both low, making bacterial sepsis less likely. A comprehensive summary of laboratory findings, including trends over time and reference ranges, is presented in Table 1.

**Table 1:** Summary of laboratory and CSF investigations showing normalization of inflammatory markers and resolution of mild abnormalities over time, consistent with a self-limiting post-infectious course. CRP: C-reactive protein; CSF: cerebrospinal fluid; CMV: cytomegalovirus; EBV: Epstein–Barr virus; PCR: polymerase chain reaction; ESR: erythrocyte sedimentation rate

Test	Reference range	Earliest/Admission	Peak/Nadir	Latest/Follow-up	Interpretation
WBC (×10⁹/L)	4.0–11.0	10.1	—	7.5	Normalised
Lymphocytes (×10⁹/L)	1.0–4.0	5.27 ↑	6.64 ↑	3.42	Initial lymphocytosis, resolved
Haemoglobin (g/L)	130–180	124 ↓	—	142	Mild anaemia, improved
Platelets (×10⁹/L)	150–400	175	—	198	Normal
CRP (mg/L)	<5	14 ↑	14–7 (early)	<1	Fell to normal
Procalcitonin (µg/L)	<0.5	0.03	—	—	Normal
ALT (U/L)	1–50	53 ↑	156 ↑	29	Transaminitis peaked then normalised
ALP (U/L)	30–130	81	—	68	Normal
Albumin (g/L)	35–50	35	32–33 ↓ (early)	43	Low early, improved
Creatinine (µmol/L)	59–104	90	—	69	Normal
ESR (mm/h)	1–5	—	22 ↑	2	Normalised
CSF opening pressure (cm H₂O)	6–25	37 ↑	—	—	Raised
CSF cells (/µL)	<3	0	—	—	Acellular
CSF protein (g/L)	0.15–0.45	0.48 ↑	—	—	Mildly raised
CSF glucose (mmol/L)	2.2–4.4	3.2	—	—	Normal
CSF virology PCR (HSV-1/2, VZV, enterovirus)	Negative	Negative	—	—	Negative
CSF CMV PCR	Negative	Negative (<500 IU/mL)	—	—	Not detected
CSF EBV PCR	Negative	Negative (<1000 IU/mL)	—	—	Not detected
Cryptococcal Ag (CSF)	Negative	Negative	—	—	Negative
CMV IgM / IgG	Negative / Negative	Positive / Positive	—	Positive / Positive(low IgG avidity)	Primary/recent CMV infection profile
CMV viral load (blood, IU/mL)	Undetectable	15,824 (high)	6,082 → 1,103	213 (low)	Falling viraemia
EBV serology	—	EBV VCA IgM+, EBV VCA IgG+, EBNA IgG+	—	—	Past infection or reactivation; see text
Antiglycolipid Ab panel	Negative	Negative	—	Negative (Follow-up)	Negative ×2
Lyme serology	Negative	—	—	Negative (Follow-up)	Negative
HIV, HBsAg, HCV	Negative	Negative	—	Negative	Negative

Lumbar puncture on day three revealed a markedly raised opening pressure, but cerebrospinal fluid was acellular with mildly raised protein and normal glucose (Table 1). CSF PCRs for common viral pathogens (herpes simplex virus, varicella-zoster virus, enteroviruses), bacterial culture, and meningitis/encephalitis panel were negative. Subsequent targeted PCR testing for CMV and EBV viral load in CSF was performed and found to be negative.

**Figure 1:**
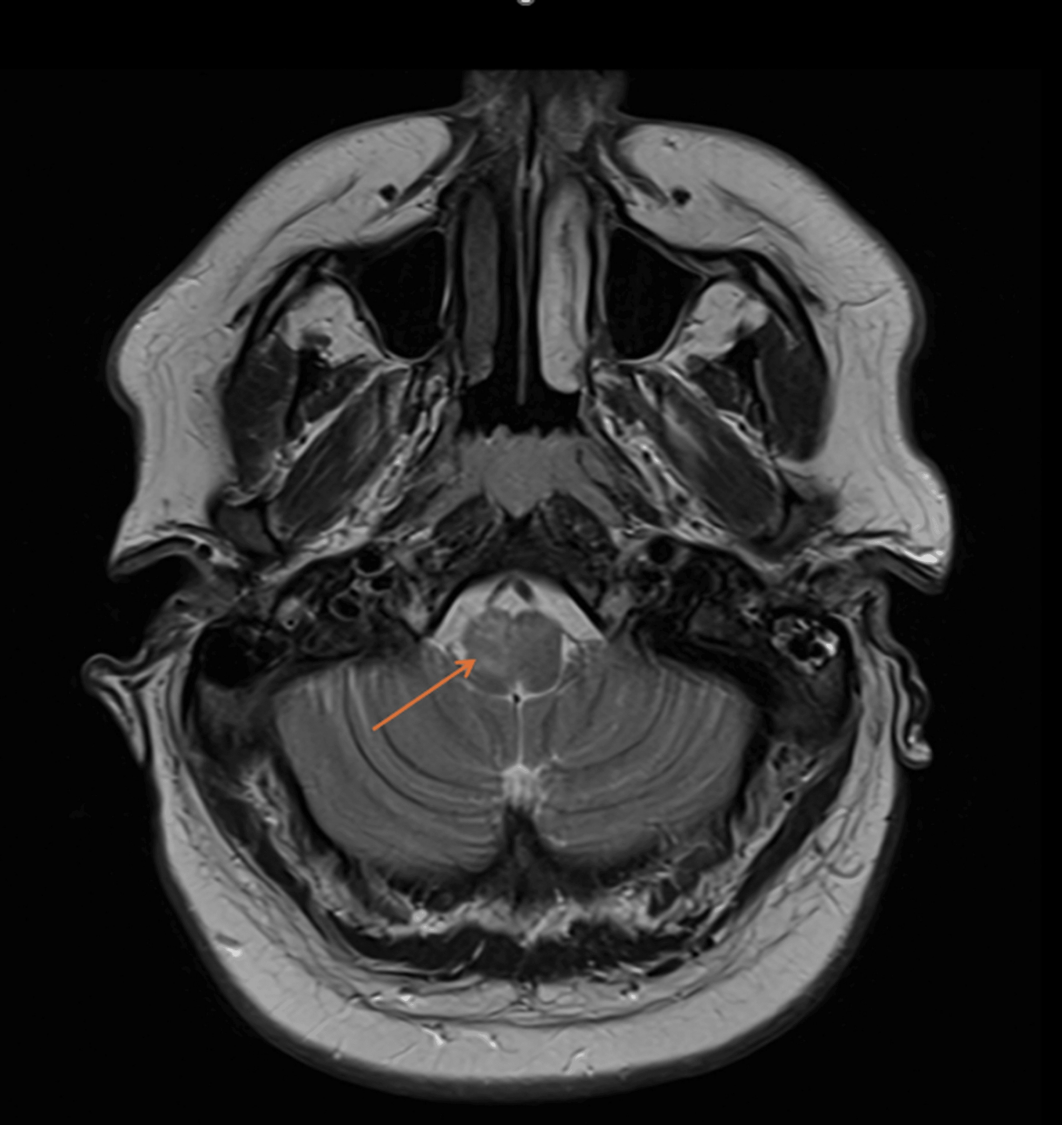
Axial T2-weighted MRI brain with contrast demonstrating a subtle area of high signal in the right medulla (arrow), without associated enhancement, consistent with inflammatory involvement in rhombencephalitis and correlating with the patient’s clinical presentation.

Neuroimaging with MRI brain and MR venography at this stage was unremarkable. A repeat MRI brain with contrast two days later demonstrated a subtle, non-enhancing T2 hyperintensity within the right medulla (Figure 1), in keeping with a focal brainstem inflammatory process. MRI spine was unremarkable, with no evidence of cord involvement.

Electroencephalography demonstrated mild diffuse encephalopathy. Nerve conduction studies and electromyography were normal, excluding a large-fibre neuropathy. Ophthalmology excluded CMV retinitis.

Serological testing was striking. CMV IgM was positive with low-avidity IgG, consistent with primary infection (Table 1). CMV viral load in blood was high initially but decreased on repeat testing. EBV VCA IgM was weakly positive, but EBNA IgG confirmed past rather than acute EBV infection. Extensive investigations for alternative infectious causes, including *Leptospira *serology, Lyme serology, and lymphocytic choriomeningitis virus (LCMV) PCR, were negative (Table 1).

Differential diagnosis

The differential at presentation was broad. Bacterial meningitis was considered but excluded on the basis of an acellular CSF, low inflammatory markers, and negative cultures (Table 1). Herpes simplex encephalitis was unlikely given the negative CSF PCR and absence of temporal lobe involvement on MRI. Venous sinus thrombosis was ruled out with a normal MR cerebral venography (MRV). Given his occupational exposure, leptospirosis and lymphocytic choriomeningitis virus were also investigated, but the results were negative (Table 1).

Transient areflexia, ophthalmoplegia, ataxia, and encephalopathy raised the possibility of a Guillain-Barré spectrum disorder, including Miller Fisher or Bickerstaff brainstem encephalitis. However, normal neurophysiology, a negative anti-glycolipid antibody panel (Table 1), and the presence of focal brainstem abnormalities on MRI (Figure 1) made this diagnosis less likely. Following multidisciplinary discussion between neurology, neuroradiology, and infectious diseases teams, the consensus was that the constellation of symptoms, imaging findings, and serological evidence was most consistent with a post-infectious CMV-associated rhombencephalitis.

Management

Empiric antibiotics and acyclovir were discontinued once cultures and PCRs were negative, and no radiological evidence supported bacterial or HSV encephalitis. Metronidazole, which had been included briefly during empiric therapy, was also stopped at this stage. The question of antiviral therapy for CMV was raised. However, given the patient’s immunocompetent status, falling viral load, absence of CSF CMV DNA, and steady clinical improvement, ganciclovir was withheld to avoid unnecessary toxicity. Management consisted of supportive care, intensive physiotherapy, and close neurological monitoring.

Outcome and follow-up

The patient made steady improvement over the subsequent week in the hospital. His level of consciousness normalised, speech became clearer, and mobility advanced from assisted ambulation to independent walking with a stick. The urinary catheter was removed as bladder function returned. At the time of discharge, mild dysarthria and imbalance persisted but were steadily improving with rehabilitation. Liver enzymes trended back towards normal, and he was referred for outpatient follow-up with infectious diseases alongside ongoing physiotherapy.

He was reviewed in the infectious diseases clinic a month later. By this stage, he had made an excellent recovery and was almost entirely back to baseline. The only residual symptom he reported was a subjective sense of unsteadiness when attempting to run at a fast pace; otherwise, his neurological examination was normal. The anti-glycolipid antibody panel that had been sent during admission returned negative (Table 1), supporting the view that a Guillain-Barré spectrum disorder was unlikely.

At follow-up, repeat blood tests were performed. The repeat anti-glycolipid antibody panel was again negative, Lyme IgM and IgG serology were negative, and CMV serology showed persistent IgM and IgG positivity with low IgG avidity (Table 1). Blood CMV viral load by PCR remained detectable but low, close to the limit of assay sensitivity. In view of his clinical recovery and reassuring investigations, he was discharged from further follow-up.

## Discussion

This case highlights the diagnostic challenges of CMV-associated CNS disease in adults without overt immunosuppression. In this population, the condition is uncommon and can present with subtle or nonspecific findings that make early recognition difficult [2,3,5-7]. Our patient had a bland CSF, an initially normal MRI, and a steadily improving clinical course, all of which complicated the diagnostic process. Similar patterns have been described in several published case reports, where the lack of early inflammatory markers delayed or obscured diagnosis [3,5,6,9,10].

A key diagnostic issue was the apparent disconnect between the patient’s significant clinical presentation and the absence of typical CSF abnormalities. This finding has been well described in post-infectious and immune-mediated presentations, where direct viral replication in the CNS is minimal or absent [10,12,13]. Normal or near-normal CSF profiles have been reported in multiple cases of CMV rhombencephalitis, underlining the importance of serology, repeat imaging, and clinical judgement rather than reliance on a single test [3,6,9]. In our case, CSF CMV PCR was negative, but serology supported recent primary infection, and repeat MRI demonstrated a medullary T2 hyperintensity correlating with the clinical syndrome (Figure 1).

The radiological findings in this case were subtle but crucial. Initial MRI was unremarkable, but repeat imaging identified a focal medullary lesion, in keeping with previously reported patterns in CMV rhombencephalitis [4,6,7,9,11]. Silva et al. described a similar dorsolateral pontomedullary lesion in a patient with positive CSF CMV PCR who improved after ganciclovir treatment [4]. Our patient, in contrast, improved without antiviral therapy, supporting a post-infectious immune-mediated mechanism rather than uncontrolled viral replication.

Another important consideration was the overlap with Guillain-Barré spectrum disorders, including Bickerstaff brainstem encephalitis and Miller Fisher syndrome. The presence of areflexia, ataxia, ophthalmoplegia, and altered consciousness could have fit these diagnoses. However, the normal neurophysiology, negative antiglycolipid antibody panel, and focal brainstem MRI changes supported post-infectious rhombencephalitis instead [12,15]. This overlap is well recognised in the literature and reflects the shared pathophysiological pathways between post-infectious immune syndromes and direct viral CNS disease.

The management of CMV CNS infection in immunocompetent adults is not standardised. Many published cases have employed ganciclovir or foscarnet with variable outcomes [2-5], but self-limiting courses without antivirals have also been documented [5,6]. In our case, the decision to withhold antiviral therapy was supported by the absence of detectable CMV DNA in CSF, declining serum viral load, and steady clinical improvement. Similar decisions have been described in cases with benign courses, where antiviral therapy was withheld when CSF CMV PCR was negative, serum viral load was declining, and clinical recovery was observed, suggesting that aggressive antiviral therapy may not be necessary when the disease is post-infectious or immune-mediated [3,5,6,9,13].

Neuroimaging findings in CMV rhombencephalitis are diverse, ranging from periventricular and cortical involvement to isolated brainstem lesions [6,7,11,14]. Our case reinforces the importance of repeating MRI in patients with progressive or unexplained neurological symptoms, as initial imaging may be normal or nonspecific. A normal CSF profile does not exclude CNS inflammation [10,13], and a high index of suspicion is needed to avoid misdiagnosis or unnecessary therapies.

Finally, this case underscores the value of multidisciplinary discussion between neurology, neuroradiology, and infectious diseases teams. Such collaborative decision-making allowed us to reach a consensus on the diagnosis and avoid unnecessary antivirals while ensuring close monitoring and appropriate rehabilitation. It also emphasises the importance of considering CMV in the differential diagnosis of acute brainstem syndromes, even in immunocompetent individuals, particularly when serology indicates recent infection.

## Conclusions

Post-infectious CMV rhombencephalitis is an uncommon but important consideration in the differential diagnosis of acute brainstem syndromes in immunocompetent adults. A normal or minimally abnormal CSF profile does not exclude CNS inflammation, particularly when the opening pressure is elevated. Serial MRI and serology can be critical in establishing the diagnosis when initial investigations are non-diagnostic. In improving patients with falling viraemia and no evidence of active CNS replication, conservative management without antiviral therapy is a reasonable and evidence-supported approach. Early multidisciplinary involvement between neurology, neuroradiology, and infectious diseases can guide appropriate diagnosis and avoid unnecessary or potentially toxic interventions.
